# Applicability of the P300 frequency pattern test to assess auditory processing

**DOI:** 10.5935/1808-8694.20130091

**Published:** 2015-10-08

**Authors:** Elisângela Barros Soares Mendonça, Lilian Ferreira Muniz, Mariana de Carvalho Leal, Alcides da Silva Diniz

**Affiliations:** a PhD Student in the Children and Adolescents Health Program - Federal University of Pernambuco - UFPE (Speech and hearing therapist at the Núcleo de Atenção ao Servidor da Gerência Regional de Educação da Mata Norte do Estado de Pernambuco na cidade de Nazaré da Mata - PE).; b PhD in Cognitive Psychology - UFPE (Professor, Department of Speech Pathology - UFPE).; c PhD from the University of São Paulo - USP (Associate Professor of Otorhinolaryngology - UFPE).; d Post-doctoral degree from the Prince Leopold Institute of Tropical Medicine - Belgium; PhD in Nutrition - UFPE (Associate Professor, Department of Nutrition UFPE).

**Keywords:** attention, electrophysiology, hearing, P300 evoked potential

## Abstract

Temporal ordering and auditory attention are important skills in information processing, being evaluated by a behavioral test, as the frequency pattern test (FPT) in temporal ordering (TO) and electrophysiological testing, as the P300 in auditory attention.

**Objective:**

To analyze the applicability of FPT and P300 as testing for auditory processing.

**Method:**

We performed an integrative literature review, with papers that met the inclusion criteria, using the MedLine, LILACS and SciELO databases, with the keywords: hearing attention, P300 evoked potential, P300 and electrophysiology, temporal ordering, processing and FPT. We found 13 papers concerning the use of the TPF and 16 regarding the use of P300.

**Results:**

The TPF was the most used test in the evaluation of TO, presented in a diotic way in individuals with language disorders, musicians, blind people, rural workers and different age groups. The P300 is used in the frequency of 1000 Hz in the frequent stimulus and 2000 Hz for the rare stimulus, applicable in individuals of both genders, different age groups, and in patients with Down syndrome, liver cirrhosis, AIDS and Sleep Apnea Syndrome.

**Conclusion:**

The FPT and P300 are efficient instruments used to assess the intended skills.

## INTRODUCTION

The Auditory Nervous System (ANS) is a highly complex system that plays a relevant role in the correct recognition and discrimination of auditory events, from the very simple - like a nonverbal stimulus, to the more complex - such as speech and language.

The brain is responsible for most of the speech auditory processing (AP), which begins in the cochlea, where the mechanical activity is transformed into nerve impulses. Physiologically, our hearing integrates three components: peripheral activity, central auditory activities and the Central Nervous System (CNS) processes^1^. When there is a break in any of these factors, there is a deficit in speech recognition.

The cerebellum is also involved in auditory processing and collaborates in several cognitive functions such as memory, language processing and linguistic operations, among others^1^. In addition to these functions, it also participates in temporal organization, maintenance and monitoring^2^, intensifying neural response and coordinating the direction of selective attention, being active in short and long term memory tests^3^.

While the peripheral auditory system receives and analyzes the auditory stimuli from the environment, the central auditory system and the brain analyze the internal representations of these acoustic stimuli and a response is programmed by the individual. The construction made from the auditory signal in order to make the information functionally useful is called auditory processing (AP) and constitutes a series of mental operations that the individual performs when dealing with information received via the sense of hearing, relying on an innate biological capacity, the maturation process, experience and the stimuli from the acoustic environment^2^. Therefore, normal hearing is necessary, but the acoustic signal has to be analyzed and interpreted in order to be transformed into a meaningful message.

AP involves a series of auditory skills such as location, detection, background information, binaural separation and others and, among them, we have temporal ordering, which can be simple when the individual identifies non-verbal sounds in the silence; and complex, when he/she identifies competitive verbal sounds, maintaining the order of presentation^4^. This ability can be analyzed by the frequency pattern test - which is behavioral, it depends on the individual's response and shows the operating mode of the subject. Another skill that makes up the AP, working in an integrated way with other skills, is auditory attention. This is made by the ability to stay focused, alert towards an auditory stimulus^5^ and can be analyzed by the P300 - an objective and physiological test capable of showing changes not yet observable in the functioning of the individual.

Other skills are involved In the assessment of temporal ordering and auditory attention, such as frequency discrimination and memory. Thus, the tests can be used together as complementing each other, bringing additional information and with greater or lesser participation of the assessed individual.

These tests are just some of the tests used to assess the ANS, specifically the complex AP, event in its struggle to elucidate its associations with other changes, but mostly with language changes.

ANS functions are influenced by the sequence of sound events that occur in time, setting the processing of temporal information^4^. The temporal AP, which serves as the basis for auditory processing is a key skill in the auditory perception of verbal and non-verbal sounds, music, rhythm and punctuation; pitch discrimination, duration and phonemes^6^.

Differences in emphasis, prosodic cues - such as pauses and speech rate, allow the listener to identify the key word and determine the semantic content^6^.

Among the temporal processing skills, we have temporal ordering, that is directly related to phonemic perception and discrimination needed to make up the phonological system of the target language^4^.

One of the leading causes of school failure among children is lack of attention^7^. This problem can be the manifestation of a number of diseases, including Attention Deficit and Hyperactivity Disorder (ADHD) and Auditory Processing Disorder (APD), among others. However, there is still no consensus on whether the difficulty in auditory attention is a component associated with the APD or if it merely reflects an isolated deficit in attention processes^7^. Auditory attention is essential for the acquisition of acoustic and phonetic aspects of language patterns - essential in the learning process of reading and writing^5^, ^7^.

Inattention is a problem that causes a person to lose or not record the information in their working memory for later processing. This disorder causes the need for more time in performing work or school tasks, since one is always seeking the information lost because of inattention, and as a result, information processing is delayed^5^. Thus, a learner with APD may demonstrate problems with understanding, discrimination and auditory memory, language deficits, background information and their learning is affected, because it depends on the degree of attention^8^. Among AP skills, hearing attention deficit is the most prevalent among school-aged children^9^.

AP assessment encompasses the listener's skill in identifying, discriminating and perceiving speech's segmental and suprasegmental aspects, and this skill is directly associated with auditory temporal aspects^10^.

Verbal and non-verbal stimuli are recommended in this type of assessment, using electrophysiological and behavioral tests to assess auditory skills^8^.

Behavioral tests are considered as key in AP diagnosis in adults and children^11^.

Among the most used behavioral tests for the detection and identification of temporal ordering are the Frequency Patterns Test (FPT) and the Duration Patterns Test (DPT)^12^.

Technically, these tests can be applied in an open field, since normative studies showed no significant difference between the right and left ears^12^.

It is observed that more than 60% of examiners in this field use the FPT, and the DPT is less frequently used^13^.

In assessing the auditory attention skill one can utilize the long-latency auditory potential, also called P300.

P300 is a positive component with a peak around 300 ms or more after the stimulus onset. It is generated using a series of sound stimuli (frequent) and different stimuli (rare) which appear at random. The test result is obtained as a function of focusing attention on the rare stimulus^14^.

The different stimulus (rare) occurs between 15 and 20% of the time, and the subject must identify it by silently counting how many times the stimulus occurs. The auditory system becomes used to hearing the frequent stimulus, and therefore fewer neurons respond to it. Rare stimuli that are heard fewer times causes the system to respond with more neurons, and therefore the curve generated by these neurons is higher than that generated by a frequent stimulus. Subtracting the rare stimulus from the frequent one we obtain the P300^15^.

The delay in P300 latency was related to a possible deficit in cognitive processing, since the triggering of this potential involves cortical areas of auditory memory, attention and perception, as well as cognition mechanisms^14^, ^15^.

The AP assessment, with the use of the mentioned tests, also aims at monitoring hearing rehabilitation through auditory training (AT), aiming at minimizing altered auditory skills, as these are necessary for the understanding of speech^12^. This technique is based on neural plasticity, which is the change in nerve cells occurring in accordance with the environmental influences and which considers young brains, such as children and adolescents with a higher plasticity which can, therefore, change^12^.

The aim of this study was to examine the applicability of the FPT and the P300 for the evaluation of temporal ordering and auditory attention, respectively, by means of a literature review.

## METHOD

**Figure 1:**
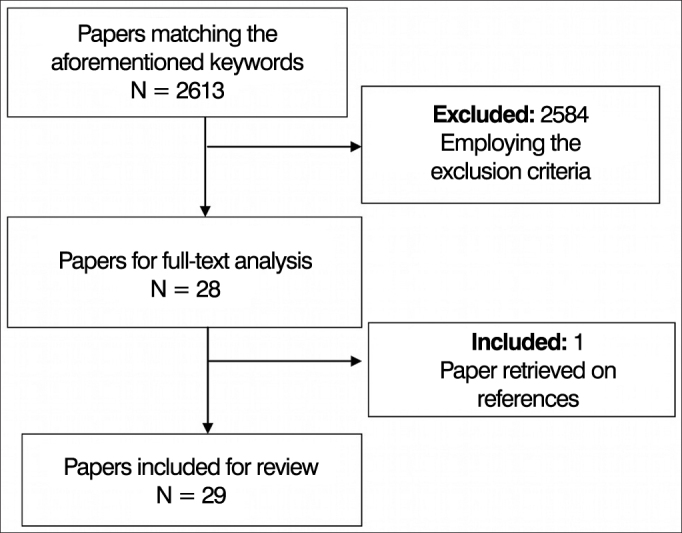
Diagram explaining the process of paper selection.

We did an integrative literature review (evidence based) to find papers indexed in the following databases: Medical Literature Analysis and Retrieval System Online (MedLine, USA), Latin American and Caribbean Health Sciences (LILACS, Brazil) and the Scientific Electronic Library Online (SciELO, Brazil). To search for papers we used the following keywords: electrophysiology and P300, auditory attention, P300 evoked potential, temporal ordering, processing and FPT. The inclusion criteria were: published and indexed full-text papers, available in the aforementioned databases in Portuguese and/or English, between 2006 and 2011, discussing FPT and P300 being used to assess temporal ordering and auditory attention skills, respectively (Figure 1).

The exclusion criteria were: papers addressing the skill in individuals with hyperactivity and attention deficit disorder, neurological diseases or injuries, psychiatric diseases, peripheral auditory disorders, stuttering, study papers involving only one case, duplicate papers in the databases and literature reviews.

We found 13 papers addressing the frequency pattern test for evaluating the temporal ordering and 16 papers using the P300 for the analysis of auditory attention.

## RESULTS

**Chart 1:**
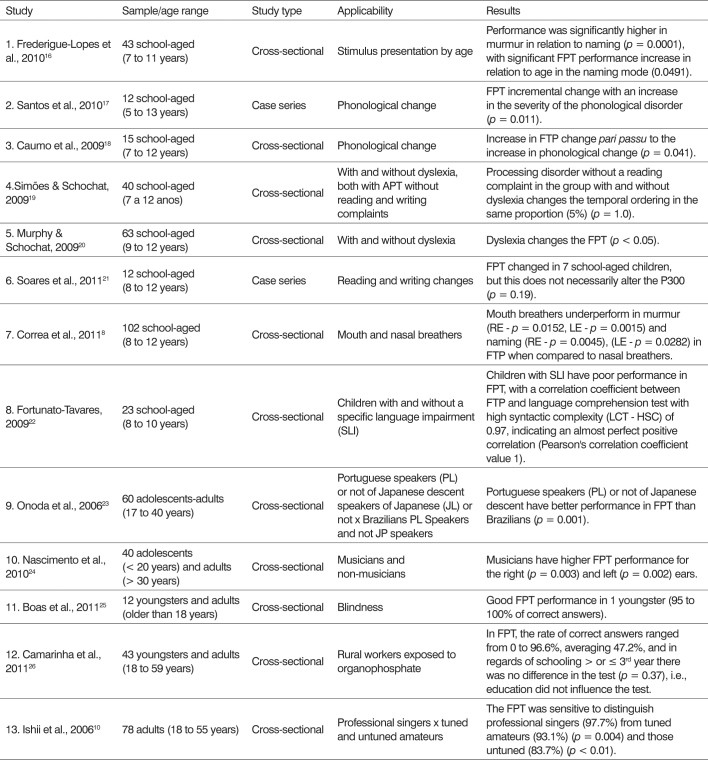
Applicability of the frequency pattern test in the assessment of temporal ordering: 2006-2011.

We noticed that the FTP can be used to evaluate temporal ordering in various situations. Most of them in individuals with language disorders, but it can also be used for musicians, blind people, rural workers, mouth breathers and in various age groups - children, adolescents, young people and adults (Chart 1).

It appears that most studies with FTP are cross-sectional, with the test being done in a soundproof booth through headphones^9^, ^10^, ^16^, ^17^, ^18^, ^19^.

FPT was employed in all studies, but in six of them, the DPT was also used to assess temporal ordering^16^, ^18^, ^20^, ^21^, ^23^, ^26^, being used in the children's Autitec^16^ or infant and adult version depending on the subject's age^17^.

One should notice that there is no uniformity in the application of the test's intensity, since it was employed at the intensity of 50 dBHL^20^, ^27^, 60 dBHL^18^, 70 dBHL^22^, ^23^, or at 50 dBHL in the three-tonal mean value^24^ or above Speech Reception Threshold (SRT)^21^.

We employed 60 stimuli in two studies^16^, ^19^, but many did not describe this figure in the methodology, although it is known that in six studies^16^, ^17^, ^20^, ^21^, ^23^, ^24^ the FPT was diotic - the stimulus was given to both ears simultaneously and in two, it was monotic^8^, ^24^. Regarding the classification for the correct answers pattern, when mentioned, we used the one recommended by Musiek (2002)^26^, ^28^ or Balen (2001)^16^, ^22^, ^27^.

On how to answer the test, either naming or murmur (humming) it appears that the studies apply one of the forms, naming^18^, ^20^, ^24^ or murmur^24^, ^25^, but most of those which described the methodology applied, used both ways or let the individual choose how best to answer^8^, ^16^, ^17^, ^23^.

There is a relationship between reading and writing and temporal processing in dyslexic individuals^19^, ^20^.

Of the 52 school-aged kids with mouth breathing and normal hearing, we found that the temporal ordering skill performance was lower than expected for their age in half of the students evaluated in both ears in the form of naming (Right ear (RE) = 29; Left Ear (LE) = 30), and in almost half of the students in the form of murmur (right ear (RE) = 22, Left Ear (RE) = 26), as well as attention and memory, since mouth breathing alters the hematological system of the individual, affecting the overall health; and daytime sleepiness can interfere with the child's attention, impairing learning^8^.

The standard test frequency is sensitive (83%) to identify auditory processing disorders resulting from brain disorders, but is not as sensitive vis-à-vis brainstem lesions (45%) or cochlear damage (12%), although it has a high specificity of 82%^29^. Recognition of the pattern as a whole would be done by the right hemisphere and the pattern sequencing by the left hemisphere, requiring an inter-hemispheric communication done by the corpus callosum. Before being decoded or sequenced by the left side it is stored in the short-term memory - and this is a brain function. The verbal response would require a subcortical neural sequence decoding of the posterior temporo-parietal area, through the intra-hemispheric tract white matter all the way to the the frontal region of the brain, within the central fissure, where the motor response would be organized and started^29^.

As for auditory attention, it is known that among the various long latency auditory evoked potentials (LLAEP), the P300 or cognitive potential is the most widely used in clinical practice and it is largely useful in the study of cognitive functions, attention and recent memory^15^.

**Chart 2:**
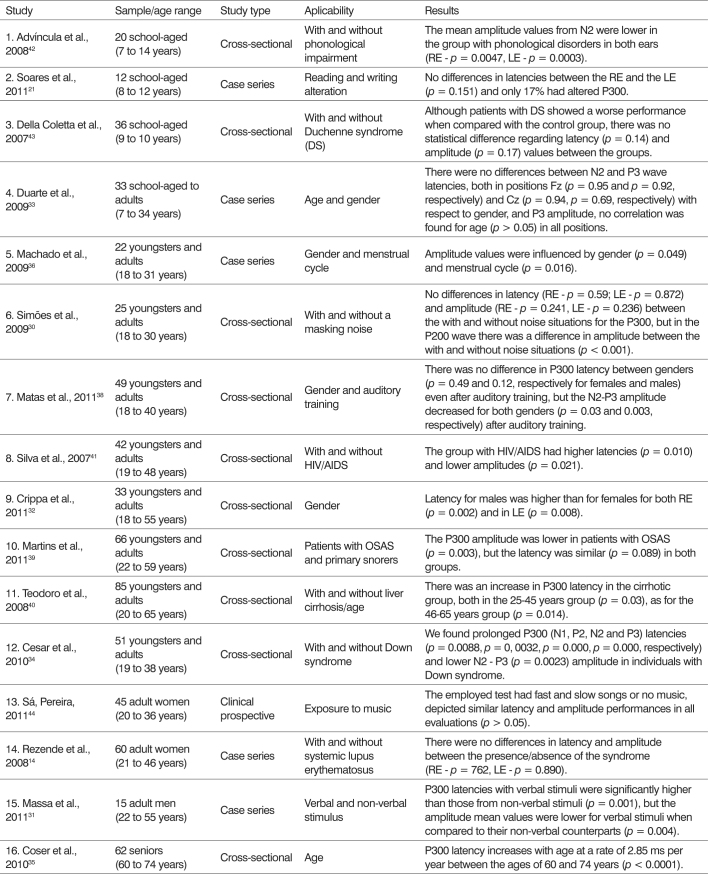
P300 applicability in the evaluation of auditory attention: 2006-2011.

It is known that the P300 has already been experienced in various situations, seeking to obtain parameters for certain age ranges^30^, ^31^, ^32^, ^33^, ^34^, ^35^, ^36^ and male latencies are larger than that of females^32^ (Chart 2).

Most studies analyze latency and amplitude; nonetheless, latency is a more reliable indicator than amplitude, since this is difficult to be changed because of attention^37^ (Chart 2).

In some studies we see that as the age of the subjects increase, P300 latency values also tend to raise^35^, ^38^, but in others the latency is stable^33^, ^38^, ^39^.

The N2-P3 amplitude has a large variability in P300, as seen in the study with its reassessment during a three-month period^40^ and in cases in which the gender and menstrual cycle period influence it^36^ (Chart 2).

In patients with sleep apnea syndrome (OSAS), there is a reduction in the P300 amplitude, suggesting cognitive dysfunction induced by auditory memory impairment^39^.

Adults with AIDS have alterations in their cognitive potential, suggesting auditory pathway involvement in cortical regions and a deficit in the cognitive processing of auditory information in this population^41^.

In general, according to the studies found in the literature, we notice that in patients with Down syndrome^34^, AIDS^41^ and phonological impairment^42^, amplitude and latency parameters were altered; however, in OSAS patients^39^, only amplitude was altered; and in those cases of liver cirrhosis without encephalopathy^40^, only latency was altered.

The equipment used in most studies for P300 implementation were the Biologic Equipment's Evoked potential System version 6.1.^32^, ^33^, ^39^ and the two-channel MK 22 Amplaid^36^, ^42^.

All the P300 tests found in the literature used the oddball paradigm, with 80% of frequent stimuli (FS) and 20% of rare stimuli (RS). The frequency used for the FS was 1000 Hz^14^, ^21^, ^30^, ^35^, ^38^, ^39^, ^40^, ^41^, ^42^, ^43^, ^44^ and 2000 Hz^14^, ^30^, ^31^, ^32^, ^33^, ^34^, ^39^, ^40^, ^42^, ^43^, ^44^ was used for the RS with four studies using 1500 Hz^21^, ^36^, ^38^, ^43^.

To implement the test, we mentioned the use of the Nuprep Abrasive Skin Gel cleaning paste on the skin^32^, the OMNI^34^ abrasive paste and the Every Per La Pulizia Della Cute^36^ - electrolytic paste for better electric current conductivity^32^, ^33^ and the electrode was secured with micropore tape^31^, ^33^, ^41^, ^42^. To facilitate relaxation during the exam, he had a quiet^21^, ^36^, ^41^, semi dark^14^, ^34^, ^36^ room, with a reclining chair^21^, ^41^, ^43^ or a stretcher^14^, ^36^, ^42^.

The electrodes were placed on the mastoid and vertex^43^, but also placed on the vertex (Cz), frontal (Fz) and mastoid (A1 and A2)^21^, ^30^, ^31^, ^33^, ^39^, ^41^, yet a considerable part of the studies used the international system^10^, ^11^, ^12^, ^13^, ^14^, ^15^, ^16^, ^17^, ^18^, ^19^, ^20^ in which the electrodes are placed on the forehead (Fz), vertex (Cz), parietal (Pz) and earlobes (A1 - left, A2 - right)^14^, ^34^, ^35^, ^36^, ^42^, ^43^, ^44^. In one study they added to the international way of electrode placement^10^, ^11^, ^12^, ^13^, ^14^, ^15^, ^16^, ^17^, ^18^, ^19^, ^20^, one electrode above the right eyebrow and one in the left corner of the eye to control the electrooculogram^40^.

It is suggested that the use of two active electrodes positioned at Fz and Cz can be considered one more resource to assist in the analysis of the P300 recording^33^.

The individual with 3A headphones^14^, ^33^, ^39^, ^44^ or TDH39^21^, ^30^, ^38^, ^41^, ^42^, was trained on the exam^14^, ^33^, ^35^, ^36^, after explaining that he should keep his attention focused on the rare stimulus, and he should count silently and raise his hand upon hearing it^36^, ^38^, ^39^, ^42^, ^44^ or count out loud^21^, ^31^, ^33^, thereby avoiding keeping the patient wide awake, being instructed to keep the eyes closed^31^, ^36^, ^42^, ^44^.

The motor act of lifting the hand associated with counting the rare stimuli is reported as being easier and so it is believed that this methodology may be adopted for patients with difficulties in performing the test only counting in sequence^36^.

The parameters used in most studies with P300 were: simultaneous binaural^32^, ^35^, ^36^, ^39^, ^40^, ^42^, monaural^14^, ^38^, ^41^, ^44^ or 100 ms rise/fall^36^ or 5 ms rise/fall^42^ e 10 ms rise/fall^43^ 20 ms plateau36,42, at 70 dBHL^14^, ^30^, ^32^, ^33^, ^43^, ^44^, 75 dBHL^31^, ^32^, ^41^ or 80 dBHL^21^, ^35^, ^36^, ^42^ with 300^14^, ^21^, ^31^, ^35^, ^38^, ^41^, ^44^ tone burst-type stimuli^21^, ^30^, ^32^, ^33^, ^35^, ^36^, ^38^, ^39^, ^41^, ^42^ at the speed of 1 s^14^, ^30^, ^32^, ^33^, ^36^, ^39^ alternating polarity^42^, rarefied^32^ or positive^31^, with a high pass of 1 Hz^38^, 2 Hz^31^ or 20 Hz^4^ and low pass at 0.5 Hz^36^, 1.5 Hz^31^ or 30 Hz^38^.

There was no latency difference between the ears^21^, ^31^ as well as between the genders^33^, ^36^.

The P300 was employed twice in two studies^14^, ^30^. However this causes fatigue and compromises the outcome of the evaluation, since it depends on attention^31^. Some authors chose to perform the electrophysiological test between 8 and 10 am^45^ or at 9 am to avoid the circadian cycle^32^.

The classifications, when referred to, were the ones recommended by Junqueira (2002)^45^, McPherson (1996)^15^ and Pfefferbaum (1984)^46^.

We notice that the different methodologies are different vis-à-vis the parameters employed and the P300 wave shape. As it turns out, the P300 latencies with verbal stimuli were significantly larger and the amplitudes were smaller for the P300 with nonverbal stimuli^31^. This probably happened because the verbal stimuli, which in this study were formed by the syllables /ba/ and /da/, are more complex, thus more difficult to hear when compared to nonverbal stimuli discrimination.

## DISCUSSION

As to the easiest test between the FPT or DPT, the FPT test is indeed considered the easiest test by 80% of the individuals^23^.

The FPT, which evaluates temporal ordering, depends on several central auditory processes, such as the recognition of the whole, inter-hemispheric transfer, linguistic qualification, sequencing of linguistic elements and evidence of memory use^47^.

One should consider that in some studies the ages of the individuals ranged from age 5 to 59 years and it is known that performance in any temporal ordering test, being it the FPT and/or DPT has a quantitative improvement in responses as age increases, especially between eight and ten years^24^, ^47^, since the corpus callosum maturation starts at seven years of age^28^ and reaches adult levels of performance on auditory processing tests at around ten or eleven years of age^24^.

We noticed that in subjects with phonological disorder^17^, ^18^, temporal ordering is changed, because the difficulty in perception of stimuli that change rapidly interferes with the phonological processing of language sounds, interfering with speech understanding and therefore in the acquisition of the target phonological system^6^ and yielding verbal language problems^4^.

The finding of a superior performance in nonverbal FPT (murmur) proves the ease of detection, recognition and retention of frequency patterns related to the execution of the murmur. Murmur does not involve memory, discrimination and awareness of the sound sequence, characterized by an imitative activity, apparently less complex^48^.

The verbal response task is more complex, indicating the need for a nervous system learning or neuromaturation. Naming as a linguistic activity, requires processes depending on more connections between thought and language^16^.

The task of temporal sequencing involves both brain hemispheres, each with a different task, but working together, regardless of the stimulated ear. The structures involved in tonal testing of auditory patterns would involve each hemisphere and the structure that connects both hemispheres is the corpus callosum. The right hemisphere would be in charge of recognizing the acoustic contour and the left one would be responsible for temporal sequencing and naming what was heard^4^, ^29^. Thus, the difficulty in the naming mode, can be explained by the need for inter-hemispheric integration (via the corpus callosum) of the stimuli in requesting a verbal response, which does not occur in the nonverbal request^28^.

Exposure to music theory and ear training are important factors in FPT performance, since they enable a greater perception in frequency discrimination, and musical practice provides this skill, making clear this association between music education and competence in frequency pattern recognition^10^, ^24^.

FPT is the most frequently used instrument in the assessment of temporal ordering, despite the existence of the Duration Pattern Test (DPT), from the age of 7, and it can improve quantitatively as we age^28^.

As for auditory attention, it is known that the auditory evoked potentials (AEP) have been characterized as an important tool in neuroscience because of their objectiveness in evaluating the structural and functional integrity of the central auditory nervous system. Besides the known clinical applications of the AEP in audiological diagnosis, intra-operative and cognitive function monitoring, its use has advantages in the assessment of language disorders, because it does not require a verbal response^38^.

The long-latency auditory evoked potentials (LLAEP) depict the cortical electrophysiological activity involved in attention, discrimination, memory, integration and decision-making skills^15^, ^30^. These potentials are associated with recorded electrical responses, generated by the thalamus, auditory cortex and cortical association areas - structures involved in discrimination, memory, attention and integration tasks^31^, ^38^, are affected by sleep, sedation and by attention to acoustic stimuli, being therefore related to attention and cognition.

In the published studies, the ages of the subjects range from 7-74 years and it is known that the P300 starts to increase in the second or third decades of life, i.e. age should be taken into account in interpreting the values obtained at different ages, since the P300 latency increases by approximately one millisecond per year of life^49^.

The decrease in P300 latency is related to increased cognitive ability; thus, following up individuals with cognitive disorders by means of the P300 can be beneficial, since this electrophysiological measure can provide information about behavioral changes of later manifestation^38^.

Attention and recent memory are dependent on stimuli discrimination, either verbal or non-verbal.

The P300 is objective; however, its analysis is extremely subjective, depending on a good clinical experience to visually detect the waves^38^.

Among the main P300 components, we list the N2 and P3 waves. N2 is a mixed - an exogenous and an endogenous - factor^45^. The exogenous factor of N2 contributes to the physical discrimination of the stimulus^37^, ^45^, ^50^ as the acoustic characteristics of the stimulus and the endogenous factor reveal attention and perception^45^, having automatic and passive responses that happen before the stimulus, elicited by the rare event, as in sound competitive situations^42^. The P3 component is an endogenous potential^46^, occurring when the individual consciously recognize a change in the auditory stimulus. These components may change when there are deficits in the attentional mechanisms.

Studies with P300 associated with attention and memory deficit report the N2 and P3 components as being sensitive to these changes. The specificity and sensitivity of this instrument is approximately 80%^50^.

Authors point out that the P300 latency increases as “targets” for discrimination are more “difficult” than the standard, i.e. the latency is sensitive to the task processing demand. In contrast, the P300 amplitude is larger for easier tasks and decreases as the task becomes more difficult^15^.

It should be noted that exposure to music can be a facilitator for the examiner to assess the P300, as it facilitates the achievement and maintenance of attention during the exam^44^, it assists in neural synchronicity and stimulates the tonotopic map of frequencies, which would facilitate the examination^40^.

We noticed the need to obtain parameters in individuals from different age groups as youngsters and adults^31^, ^32^, ^33^, ^36^, ^38^, and for healthy older adults^35^ and we realized that the P 300 needs to be further studied.

The application of P300 in the evaluation of auditory attention is very common, however it is still an object to obtaining parameters as we seek to evaluate it in different age groups, healthy subjects and in cases with language disorders and there is no single methodology for use.

## FINAL REMARKS

The FPT is an instrument most often used to assess temporal ordering, generally used with a CD Player connected to the audiometer, receiving the stimuli through a headphone, being applicable to individuals with phonological disorders, mouth breathing, language disorders and rural workers, performing well in blind adults and Japanese descendants - as a second language facilitates sound frequency pattern recognition.

The P300 can be used with various parameters, and latency is the best indicator for analyzing auditory attention, being applicable in patients with Down syndrome, AIDS, phonological disorder, OSAS and liver cirrhosis, in subjects of both sexes and different age groups.

Exposure to music is a factor that can help improve temporal ordering and P300 use to assess auditory attention, because this characteristic favors auditory memory training and frequency discrimination, abilities that help in processing the investigated skills.
